# Trust-Aware Domain Adaptation Using Physics-Guided Reliability Learning for Cross-Condition Fault Diagnosis of Milling Machines

**DOI:** 10.3390/s26144473

**Published:** 2026-07-14

**Authors:** Saif Ullah, Soonhyun Lim, Jong-Myon Kim

**Affiliations:** Department of Electrical, Electronics and Computer Engineering, University of Ulsan, Ulsan 44610, Republic of Korea; saifuou@mail.ulsan.ac.kr (S.U.); lsh0981@mail.ulsan.ac.kr (S.L.)

**Keywords:** fault diagnosis, milling machine, domain adaptation, condition monitoring, vibration signals

## Abstract

Reliable fault diagnosis of milling machines under varying operating conditions remains challenging due to distribution shifts caused by speed variations, nonstationary dynamics, and limited labeled data in target domains. Conventional domain adaptation methods often assume equal reliability across samples and neglect the varying physical consistency of signals collected under different conditions. To address this limitation, this study proposes trust-aware domain adaptation network for cross-domain fault diagnosis that integrates physics-guided reliability estimation with deep representation learning. In the proposed framework, physically interpretable global and local features are first extracted from multi-channel vibration signals using energy, spectral, nonlinear, and impulsiveness descriptors. A dedicated Physics Trust Network is then introduced to estimate per-sample trust scores that quantify the physical reliability of each signal based on its physics feature consistency. These trust scores are explicitly embedded into representation learning through a trust-weighted feature encoder, ensuring that physically reliable samples contribute more strongly to the learned latent space. To address distribution mismatch between source and target conditions, a trust-weighted covariance alignment strategy is introduced, enabling domain adaptation to be guided by reliable samples instead of treating all data equally. In this way, the model simultaneously learns discriminative, transferable, and physically consistent features. The entire framework is trained end-to-end using labeled source data and unlabeled target data, enabling effective knowledge transfer under cross-speed conditions. Extensive experiments on a real milling machine dataset collected at different spindle speeds demonstrate that the proposed framework achieves an average accuracy of 98.07%, performing better than two recent state-of-the-art domain adaptation approaches by a significant margin. Ablation experiments further confirm that reliability estimation, trust-weighted representation learning, and trust-guided alignment each contribute independently to performance improvement.

## 1. Introduction

Milling machines play a central role in present-day manufacturing sectors, including aerospace, aeronautical engineering, and the automotive industry. These systems shape raw metallic workpieces into precise components through material removal processes [1]. Due to continuous operation under high rotational speeds and substantial mechanical loads, milling machines are highly susceptible to degradation and failure of critical elements such as bearings, gears, and cutting tools. Faults in milling machines are broadly classified into electrical and mechanical categories, with mechanical issues comprising nearly 57% of all reported failures. Within this group, bearing-related defects are the most prevalent, accounting for approximately 42% of mechanical failures [2]. Moreover, defects in gears and bearings significantly disrupt spindle dynamics and operational stability [3]. Failures of cutting tools alone contribute to nearly 20% of unexpected machine downtime and associated economic losses [4]. Overall, failures in mechanical components lead to production interruptions, increased maintenance expenditures, safety risks including possible injuries or loss of life, and a decline in machining performance such as reduced cutting speeds [5]. These serious consequences motivate the development of intelligent fault diagnosis approaches capable of accurately detecting and distinguishing defects in critical milling machine components, including gears, cutting tools, and bearings.

In recent years, intelligent condition monitoring methods have significantly transformed manufacturing practices by improving operational efficiency, sustainability, and economic performance [6,7]. This progress has been driven by the integration of sensors on milling machines, enabling continuous real-time data acquisition combined with artificial intelligence for automated analysis and decision making [8]. Within the existing body of literature, condition monitoring techniques for milling machines are generally divided into two principal categories: direct and indirect approaches. Direct condition monitoring techniques focus on inspection of machine components, primarily relying on machine vision systems to detect variations in tool geometry, measure flank wear width, estimate wear area and volume, and assess the quality of the machined surface. Direct monitoring approaches provide better accuracy and repeatability compared to manual inspection; however, they suffer from several practical limitations. These methods often require extended machine stoppages, are sensitive to cutting fluids, demand physical access to all machine components, and rely on controlled lighting environments. As a result, they are generally less attractive than indirect nondestructive testing approaches. Indirect techniques acquire physical responses from milling machines using sensors, producing measurable signals that are subsequently analyzed through advanced signal processing and artificial intelligence for reliable fault diagnosis [9]. Commonly reported indirect sensing modalities include electrical current signals [10], cutting force measurements [11], vibration responses [12], and acoustic emission signals [13].

Over time, a wide range of techniques has been developed to analyze these signals, primarily categorized into time domain (TD) [14], frequency domain (FD) [15], and time frequency domain (TFD) approaches [16,17]. Time domain analysis has been widely used for fault detection through statistical indicators such as the root mean square (RMS) value [18]. Frequency domain techniques focus on variations in spectral content and frequency shifts, but they are mainly effective for stationary signals and show limited performance when applied to the nonstationary signals typically generated during milling processes [19]. To address this limitation, time frequency analysis methods, including wavelet-based techniques and empirical mode decomposition, have been introduced to better capture the transient and nonstationary characteristics of signals [20]. Despite their advantages, these conventional signal processing approaches remain constrained by high sensitivity to noise and limited adaptability when dealing with complex nonstationary signal behavior.

Recent progress in deep learning has shown that data-driven models can automatically learn discriminative features from raw inputs that are highly effective for fault recognition tasks [21]. Among these models, convolutional neural networks have demonstrated strong capability in extracting spatial patterns from time frequency representations such as scalograms, leading to accurate identification of complex fault conditions. However, these kinds of architectures often face limitations when capturing the temporal relationships that naturally exist in signals. To overcome this limitation, more sophisticated network designs have been introduced, including hybrid frameworks that combine convolutional neural networks with Long Short-Term Memory networks to model sequential dependencies [22,23], as well as architectures with residual connections that facilitate deeper learning and improve representation accuracy for complex fault behaviors [24,25].

In parallel, several studies have addressed the issue of limited labeled data in industrial fault diagnosis. Su et al. [26] proposed a small sample fault diagnosis framework for wind turbine gearboxes using optimized generative adversarial networks to generate synthetic fault data and improve generalization performance. Fan et al. [27] presented a metric-based learning approach with zero shot capability, allowing effective fault classification even for previously unseen categories. In a related effort, Fan et al. [28] developed a digital twin-assisted degradation evaluation strategy for bearing cages by combining physics-based modeling with data-driven analysis to support predictive maintenance.

Multi sensor fusion has become a powerful strategy in fault diagnosis, as it improves accuracy and reliability by combining information from different sensor types. By integrating complementary data sources, this approach enables more stable and precise fault detection, even under harsh and noisy operating conditions [29]. Huo et al. [30] proposed a diagnostic framework that integrates vibration signals with infrared thermography data for rotor bearing systems. Their study demonstrated that the fused representation significantly improves robustness and fault identification performance, particularly in environments affected by noise. Deep learning models were utilized together with Dempster Shafer evidence theory at the decision level, showing that multi sensor integration outperforms single sensor-based diagnosis. In another study, Chen and Li developed a bearing fault diagnosis method based on multi sensor data fusion using a sparse autoencoder and a deep belief network [31]. Features extracted from time domain and frequency domain signals collected through multiple accelerometers were fused using the sparse autoencoder to achieve more reliable diagnostic results. Similarly, Hoang and Kang introduced a bearing fault diagnosis framework that combines deep belief networks with Dempster Shafer evidence theory, demonstrating that multi sensor data integration enhances the detection of early-stage bearing faults. Beyond neural network-based feature extraction, Guan et al. [32] proposed the 2MNet framework, which performs joint fusion of multi sensor and multi scale features for rolling bearing fault diagnosis. In addition, Mian et al. [33,34] investigated the combined use of infrared thermography and vibration signals to diagnose misalignment, imbalance, and eccentricity faults in rotating machinery. Despite its performance advantages, multi sensor fusion involves a tradeoff associated with increased hardware complexity and implementation cost.

To address the above limitations, this work is motivated by the practical challenge that milling machines vibration signals collected under different spindle speeds often show strong distribution shifts, nonstationary behavior, and unequal signal reliability. Existing domain adaptation methods mainly focus on aligning source and target feature distributions; however, they usually assume that all samples are equally informative during adaptation. In real milling operations, this assumption is not always valid because some vibration segments may be affected by noise, weak fault signatures, transient cutting behavior, or unstable machining dynamics. If such unreliable samples are treated equally, they may distort the learned feature space and cause negative transfer between operating conditions.

Therefore, this study proposes a trust-aware physics-informed domain adaptation framework for reliable fault diagnosis of milling machines under varying spindle speeds and operating conditions. The key motivation is to make domain adaptation reliability-aware by allowing physically consistent samples to contribute more strongly to representation learning and feature alignment, while reducing the influence of uncertain or inconsistent samples. The method constructs a physics-consistent feature representation by integrating interpretable global and local vibration descriptors, enabling comprehensive characterization of fault behavior beyond conventional feature fusion strategies. A Physics Trust Network is introduced to estimate the reliability of each signal sample, allowing informative and physically consistent signals to guide the learning process while reducing the influence of unreliable measurements. Guided by these reliability estimates, the framework performs domain adaptation between operating conditions and handles the distribution shifts caused by speed variation and nonstationary dynamics. As a result, the proposed approach enables stable unsupervised knowledge transfer and improves diagnostic reliability in real industrial environments.

It should be noted that the proposed framework does not embed a complete first-principles dynamic model of the milling process. Instead, physical knowledge is incorporated through vibration descriptors that have clear mechanical interpretation, including energy distribution, spectral shift, impulsive response, and nonlinear signal complexity. These descriptors are used not only as input features but also to estimate sample reliability, which subsequently guides feature learning and domain alignment. The main contributions of this work are as follows:

(1) A trust-aware physics-guided domain adaptation network is proposed for cross-speed fault diagnosis of milling machines, enabling reliable knowledge transfer under spindle speed variation and nonstationary machining conditions.

(2) A physics-consistent feature representation is developed by integrating interpretable global and local vibration descriptors covering energy, spectral, nonlinear, and impulsiveness characteristics, allowing comprehensive characterization of tool health behavior.

(3) A Physics Trust Network is introduced to estimate per sample reliability, providing a principled mechanism to evaluate the physical consistency of signals instead of assuming equal importance of all samples during adaptation.

(4) A reliability-driven adaptation strategy is designed in which trust scores guide both feature learning and domain alignment, allowing reliable samples to dominate knowledge transfer and reducing negative transfer between operating conditions.

(5) Experimental and ablation studies on real milling machine data demonstrate improved diagnostic accuracy, robustness to speed variation, and better interpretability compared with conventional and recent domain adaptation approaches.

The structure of this paper is organized as follows: Section 2 explains the experimental setup as well as the data acquisition. Section 3 describes the detailed proposed methodology. Section 4 details the results and discussion. Finally, Section 5 concludes the study with future recommendations.

## 2. Experimental Setup and Data Acquisition

**Figure 1:**
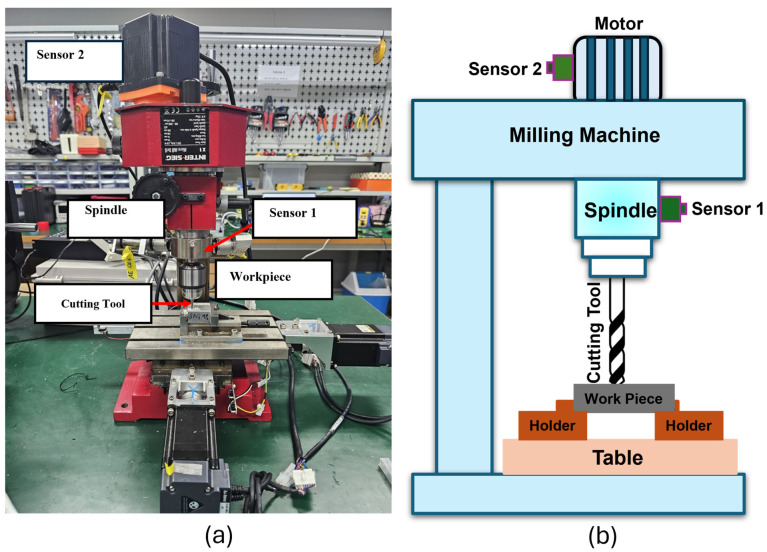
Experimental setup: (**a**) real and (**b**) schematics.

The milling machine dataset used in this study was acquired from a dedicated MCT experimental testbed as shown in Figure 1. It is designed to capture vibration signals corresponding to different machine health conditions. The experimental setup consisted of a motor-driven shaft coupled to a 16-tooth gear, followed by a secondary shaft carrying 32-tooth and 30 tooth gears. The spindle was mechanically linked to the 30-tooth gear and equipped with a two-flute end mill cutting tool. This configuration enabled controlled transmission of torque from the motor to the spindle while allowing vibration responses from critical rotating components to be measured.

The machining system used in this study was an INTER SIEG X1 Micro Mill Drill (Inter Sieg, Bremen, Germany) constructed from cast iron and comparable in operation to a compact pillar drilling machine. Vibration signals were measured using accelerometer sensors mounted on the milling machine through adhesive fixation. Data acquisition was carried out using National Instruments (Austin, TX, USA) NI 9234 module details provided in Table 1, controlled via custom Python 3.10.0-based software developed at the Ulsan Industrial Artificial Intelligence Laboratory. The vibration signals were sampled at a frequency of 25,600 Hz to capture detailed dynamic responses during machining. Prior to the experiments, sensor functionality was verified to ensure accurate signal acquisition. Two vibration sensors were used, with the primary sensor attached to the spindle to capture tool and rotating component related dynamics, while a secondary sensor mounted on the motor served to identify nontarget vibrations and suppress background noise.

**Figure 2:**
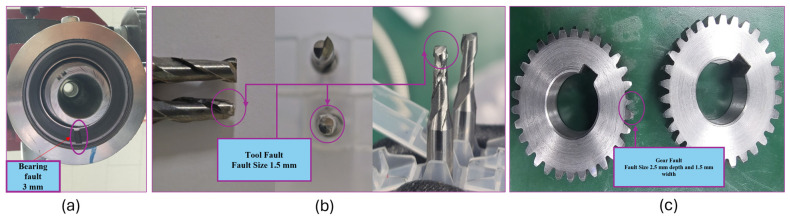
Faults: (**a**) bearing fault, (**b**) tool fault, and (**c**) gear fault.

Data collection was initiated under healthy operating conditions of the milling machine. In accordance with ISO 8688 2 guidelines [35], tool wear is defined by an average flank wear threshold of 0.3 mm. Since unexpected tool damage may occur even at early wear stages during the machining of hard materials, an initial average flank wear of 0.3 mm was artificially introduced to represent controlled fault conditions for the study. Bearing faults were created by inducing localized damage with an approximate depth of 3 mm on the bearing supporting the cutting tool. To replicate transmission-related faults, a minor portion of a gear tooth involved in torque transfer between the motor and spindle was removed, generating an incipient gear defect. These defect pictures are illustrated in Figure 2.

**Figure 3:**
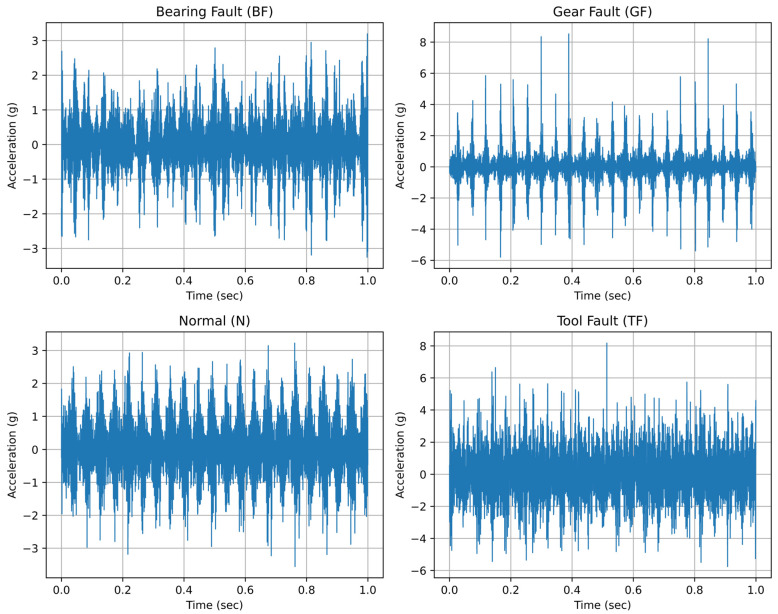
Vibration signals of different fault types and normal condition at 1320 rpm.

**Figure 4:**
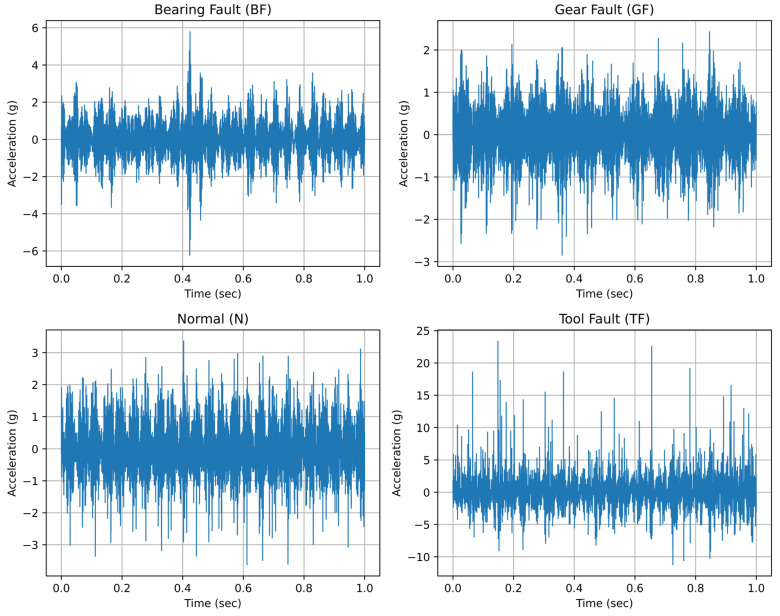
Vibration signals of different fault types and normal condition at 1440 rpm.

Vibration signals were continuously acquired throughout the machining process to monitor these induced faults and evaluate the overall machine condition. All data was recorded during active milling operations only, ensuring consistency across classes. The spindle and motor operated at fixed speeds of 1320 rpm and 1440 rpm, respectively, with a constant feed rate of 0.4 mm/s. Vibration signals were sampled at 25.6 kHz using an NI 9234 data acquisition module, with sensors mounted on the motor and spindle to capture dynamic responses associated with tool, gear, and bearing behavior. This dataset therefore comprises four classes: normal, bearing fault, gear fault, and tool fault. It provides a reliable basis for evaluating vibration-based fault diagnosis methods under realistic machining conditions. A comprehensive summary of the dataset structure, including class distribution and operating conditions, is provided in Table 2 for clarity. Vibration signals obtained under operating conditions of 1320 rpm and 1440 rpm are shown in Figure 3 and Figure 4 respectively.

It should be noted that the four datasets listed in Table 2 are independent acquisition batches rather than duplicate divisions of the same recording. Datasets 1 and 2 were collected at different acquisition times under the same nominal spindle speed of 1320 rpm, while Datasets 3 and 4 were collected at different acquisition times under the same nominal spindle speed of 1440 rpm. Although the nominal speed is the same within each pair, the repeated acquisition sessions may still contain natural variations caused by machining dynamics, sensor contact conditions, cutting interaction, and time-dependent operational fluctuations. Therefore, these datasets provide independent repeated measurements under the same speed as well as cross-speed conditions for domain adaptation evaluation.

To ensure a clear unsupervised domain adaptation protocol, each cross-speed transfer task was constructed using one dataset as the labeled source domain and another dataset as the target domain. The complete source dataset was used as labeled source-domain training data. The target dataset was split using a stratified 80:20 ratio. Specifically, 80% of target-domain samples were used as unlabeled target-domain training data for domain alignment, while the remaining 20% were used as the labeled target-test set for final evaluation only. The target-test labels were not used during model training or domain adaptation.

Each vibration sample consisted of two channels and contained 25,600 data points per channel, corresponding to a one-second signal segment at the sampling frequency of 25.6 kHz. No overlapping windows were used to generate the samples. During local physics-feature extraction, each signal sample was divided into non-overlapping patches of 256 samples; however, these patches were used only for feature computation and did not increase the number of training or testing samples.

## 3. Proposed Methodology

**Figure 5:**
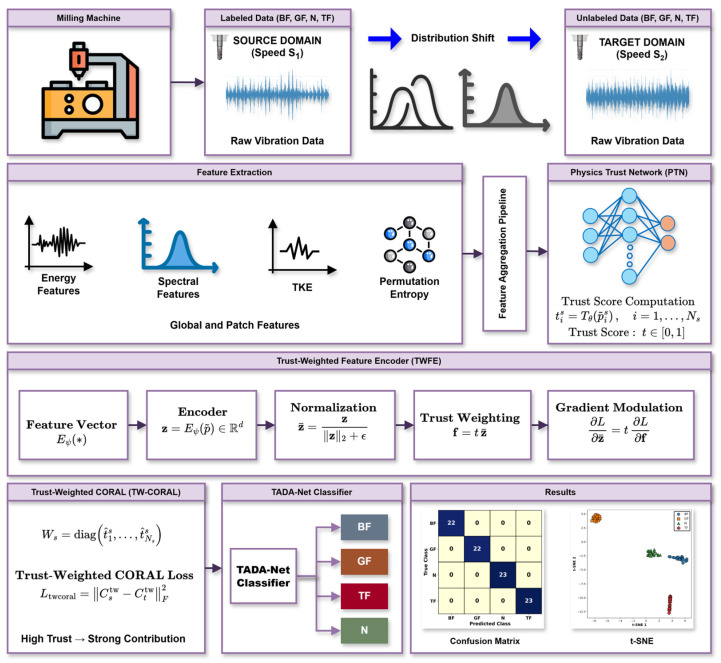
Proposed method workflow.

This study presents a physics-guided trust-aware domain adaptation framework designed for reliable fault diagnosis of milling machines under cross speed operating conditions. Variations in spindle speed introduce distribution shifts and inconsistent signal quality, which can degrade the effectiveness of conventional adaptation methods that treat all samples equally. To address this issue, the proposed framework incorporates signal reliability into the learning process. First, physically interpretable global and local features are extracted from multi sensor vibration signals to characterize energy, spectral, impulsive, and nonlinear behavior of the tool condition. A Physics Trust Network then estimates a reliability score for each sample by evaluating the physical consistency of these features. The obtained trust scores are embedded into feature learning so that reliable samples exert stronger influence on the learned representation while unreliable signals are suppressed. The trust-weighted representations are subsequently aligned between source and target domains using a covariance-based adaptation strategy, and the aligned features are used for final fault classification. The overall workflow of the proposed framework is illustrated in Figure 5, and each step is described in detail below.

**Step 1: Cross-speed domain learning setup and reliability-aware objective**

Milling machine vibration dataset is collected under two spindle speeds. The source domain contains labeled samples collected at speed $r_{s}$ and the target domain contains samples collected at speed $r_{t}$. Due to speed variation, load changes, and machining dynamics, the data distributions differ even when the fault classes are the same. This induces a distribution shift between source and target domains.

Let the source domain be mathematically represented as:

(1) $$D_{s}=\{\left(x_{i}^{s},y_{i}^{s}\right){\}}_{i=1}^{N_{s}}$$

In Equation (1), $x_{i}^{s}\in R^{C\times T}$ is a multi-channel vibration signal segment with C=2 channels and length T, and $y_{i}^{s}\in \{1,\ldots ,K\}$ is the fault label for K=4 classes (BF, GF, N, TF). The target domain is split into unlabeled training and labeled testing as given in Equation (2).

(2) $$D_{t}^{u}=\{x_{j}^{t}{\}}_{j=1}^{N_{t}},\;\;\;D_{t}^{test}=\{(x_{m}^{t},y_{m}^{t}){\}}_{m=1}^{N_{test}}$$

The goal is to learn a classifier h(∗) that performs well on the target-test set although the model is trained using labels only from the source domain and uses target training samples without labels. Formally, we want to minimize the expected target error.

(3) $$\epsilon_{t}(h)=E_{(x,y)∼D_{t}^{test}}\left[I(h(x)\neq y)\right]$$

While labels from $D_{t}^{u}$ are unavailable during training, a major challenge is that not all vibration samples are equally reliable. Samples may be corrupted by noise, transient events, or weak fault signatures. If we adapt domains by treating every sample equally, unreliable samples can dominate the alignment and degrade performance. Therefore, we propose a trust-aware model where each sample receives a physics-based trust score t∈[0,1] that controls its contribution to representation learning and domain alignment. This becomes the central idea that converts domain adaptation into a reliability weighted learning problem.

**Step 2: Feature construction from multi-channel milling vibration signals**

Instead of feeding raw signals directly into domain alignment, we first construct physically interpretable descriptors from each vibration sample. Let $x\in R^{C\times T}$ be one sample where C=2. For channel C, denote $x_{C}(n)$ for n=1,…,T. The first descriptor is the RMS energy, defined as:

(4) $$E_{c}=\sqrt{\frac{1}{T}\sum_{n=1}^{T}x_{c}^{2}(n)+\varepsilon }$$

In Equation (4), ε is a small constant to avoid numerical issues. To capture frequency behavior in milling, we estimate the power spectral density using Welch PSD, $P_{c}(f)$. From the PSD we compute band energy ratios over predefined frequency bands $B_{b}=[f_{b}^{lo},f_{b}^{hi}]$ as below.

(5) $${\mathrm{BER}}_{c,b}=\frac{\int_{f_{b}^{lo}}^{f_{b}^{hi}}P_{c}(f)df}{\int_{0}^{f_{s}/2}P_{c}(f)df+\varepsilon }$$

This quantifies how vibration energy concentrates in low, mid, and high bands, which changes with tool wear and fault induced impacts. Normalized spectral centroid is also calculated using Equation (6).

(6) $${\mathrm{SC}}_{c}=\frac{\int_{0}^{f_{s}/2}fP_{c}(f)df}{\int_{0}^{f_{s}/2}P_{c}(f)df+\varepsilon }*\frac{1}{f_{s}/2}$$

This capture shifts toward higher frequency content as cutting stability degrades. To model impulsiveness, the Teager Kaiser Energy Operator is calculated as given in Equation (7).

(7) $$\Psi_{c}(n)=x_{c}^{2}(n)-x_{c}(n-1)x_{c}(n+1)$$

From $\Psi_{c}(n)$ we compute means absolute value and variance which characterize impulsiveness and transient behavior caused by tool impacts such as:

(8) $$\mu_{\Psi_{c}}=\frac{1}{T-2}\sum_{n=2}^{T-1}|\Psi_{c}\left(n\right)|,\sigma_{\Psi_{c}}^{2}=\frac{1}{T-3}\sum_{n=2}^{T-1}(\Psi_{c}\left(n\right)-{\overset{ˉ}{\Psi }}_{c}{)}^{2}$$

To quantify complexity, we compute permutation entropy $H_{c}$ based on ordinal patterns with embedding dimension M using Equation (9) as:

(9) $$H_{c}=-\sum_{m=1}^{M}p_{m}log(p_{m}+\varepsilon ),H_{c}^{norm}=\frac{H_{c}}{log(M)}$$

For each channel we form an 8-dimensional vector $g_{c}$. Then we aggregate across channels to form a 16-dimensional global physics vector, mathematically given as:

(10) $$p=[\mathrm{mean}(g_{1},g_{2}),\;\mathrm{std}(g_{1},g_{2})]\in R^{16}$$

This physics vector is the input to trust modeling and the downstream encoder. Finally, to ensure fair adaptation, we normalize using source-only statistics. If $\mu_{s}$ and $\sigma_{s}$ are the source mean and standard deviation across $p_{i}^{s}$, Equation (11) is given as:

(11) $$\tilde{p}=\frac{p-\mu_{s}}{\sigma_{s}+\varepsilon }$$

This avoids target information leakage and preserves the integrity of the unsupervised adaptation setting.

**Step 3: Physics Trust Network for sample-wise reliability estimation**

Even with physically interpretable descriptors, not all samples are equally reliable. For example, a milling tool vibration signal may be affected by chatter, measurement disturbances, or weak fault excitation. If such samples dominate training, the model can learn spurious patterns and align domains in a misleading direction. To solve this, we introduce a Physics Trust Network that assigns a trust score to every sample from its physics feature vector representing its physical reliability for alignment and classification.

The physical meaning of the trust score can be interpreted from the vibration descriptors used as input to the Physics Trust Network. A high trust score indicates that the sample has physically consistent vibration characteristics, such as stable energy behavior, meaningful spectral distribution, clear impulsive response related to mechanical impacts, and nonlinear complexity consistent with the corresponding operating condition. For example, a reliable fault-related sample may contain distinct impulsive or spectral patterns caused by bearing, gear, or tool defects, while a reliable normal sample may show comparatively stable vibration energy and fewer irregular transient components.

In contrast, a low trust score indicates that the sample is physically ambiguous or less reliable for adaptation. Such samples may be affected by measurement noise, weak fault excitation, transient cutting disturbances, chatter-like vibration, sensor contact variation, or background vibration. These effects can distort RMS energy, shift the spectral centroid irregularly, produce broadband spectral fluctuations, alter Teager–Kaiser energy responses, or increase nonlinear complexity measured by permutation entropy. Therefore, low-trust samples are not removed from training, but their influence is reduced during trust-weighted feature encoding and trust-weighted CORAL alignment.

Let $\tilde{p}\in R^{16}$ be the normalized global physics vector. The network maps a 16-dimensional physics vector to a scalar trust score. The trust network is a parametric mapping $T_{\theta }(*)$ defined as a multilayer perceptron as:

(12) $$t=T_{\theta }(\tilde{p})=\sigma \left(w_{3}^{⊤}\phi \left(W_{2}\phi \left(W_{1}\tilde{p}+b_{1}\right)+b_{2}\right)+b_{3}\right)$$

In Equation (12), ϕ(∗) is a nonlinear activation; in this study ReLU, and σ(∗) is the sigmoid function. This ensures $t\in \left(0,1\right)$ and can be interpreted as continuous reliability weight. Conceptually, t is expected to be high when physics features indicate stable cutting dynamics or clearly distinguishable fault signatures, and low when the feature pattern is ambiguous or physically inconsistent. It is important to clarify how the Physics Trust Network is trained. In this study, no manually annotated sample-level reliability labels are available; therefore, $T_{\theta }$ is not trained using a separate supervised trust-label loss. Instead, the trust score is treated as a latent reliability weight that is learned end-to-end together with the feature encoder and classifier. The training is supervised only with respect to source-domain fault labels through the classification loss, while the target-domain data remain unlabeled and contributes through the unsupervised domain alignment loss. Thus, $T_{\theta }$ is optimized indirectly through the final task objective rather than through direct trust-score supervision. We then collect trust scores for source and target:

(13) $$\;t_{i}^{s}=T_{\theta }({\tilde{p}}_{i}^{s}),i=1,\ldots ,N_{s}$$

(14) $$t_{j}^{t}=T_{\theta }({\tilde{p}}_{j}^{t}),j=1,\ldots ,N_{t}$$

For numerical stability, we can normalize trust scores within a batch:

(15) $${\hat{t}}_{i}=\frac{t_{i}}{\sum_{k}t_{k}+\varepsilon }$$

This normalization is important later when trust is used inside covariance alignment so that the scale does not depend on batch size. This normalization is applied separately to the source and target batches during training.

**Step 4: Trust-weighted feature encoding and trust modulated representation learning**

After computing the trust score, a discriminative representation is learned using a neural encoder, but the encoder is explicitly modulated by trust. Let $E_{\psi }(*)$ be a feature encoder with parameters ψ that maps physics features to a latent representation.

(16) $$z=E_{\psi }(\tilde{p})\in R^{d}$$

In Equation (16), in the proposed pipeline d=64. To stabilize the feature scale before trust modulation, the latent feature vector is first L2-normalized as:

(17) $$\bar{z}=\frac{z}{∥z{∥}_{2}+\varepsilon }$$

In Equation (17), ϵ is a small constant added to avoid division by zero. The trust score is then applied to the normalized latent feature representation:

(18) $$f=t.\bar{z}$$

In Equation (18), t is the scalar trust score and is broadcast across all feature dimensions. It is important to note that normalization is applied before trust weighting; therefore, the scalar trust score is not removed by the normalization operation. Instead, t directly scales the final representation f, which affects both the classifier gradient updates and the feature statistics used for domain alignment. Hence, low-trust samples produce lower-magnitude feature representations, while high-trust samples contribute more strongly to classification learning and domain alignment. This ensures that the encoder output is scale-stabilized before trust modulation, while the trust score still controls the effective contribution of each sample during classification and alignment. Now we attach a classifier $C_{\omega }(*)$ that outputs logits as below.

(19) $$a=C_{\omega }\left(f\right)\in R^{K},\;\;\;\hat{y}=\mathrm{softmax}(a)$$

In Equation (19), K=4 corresponds to the fault classes. For source samples, we compute cross entropy as given in Equation (20).

(20) $$L_{cls}=-\frac{1}{N_{s}}\sum_{i=1}^{N_{s}}\sum_{k=1}^{K}I(y_{i}^{s}=k)log\left({\hat{y}}_{i,k}^{s}\right)$$

The trust modulation also changes the optimization behavior because the gradient of the normalized latent representation is scaled by the trust score:

(21) $$\frac{\partial L}{\partial \bar{z}}=t\cdot \frac{\partial L}{\partial f}$$

**Figure 6:**
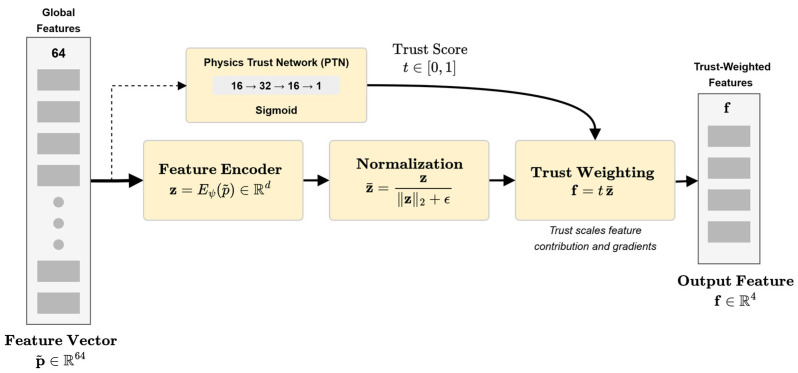
Schematic representation of Trust-weighted feature encoding.

Similarly, because CORAL alignment is computed using the trust-modulated representation f, low-trust samples contribute less to the source and target feature statistics, while high-trust samples dominate the alignment process. Thus, low-trust samples naturally produce smaller updates, which is desirable when samples are physically unreliable. In milling machine fault diagnosis, this is valuable because signal quality can vary due to cutting engagement, material inhomogeneity, chatter onset, and sensor contact conditions. The schematic of this step is represented in Figure 6.

**Step 5: Trust-weighted CORAL alignment for unsupervised domain adaptation**

To adapt the source learned representation to the target domain, this study proposes second order statistics alignment. CORAL aligns covariances of features. However, standard CORAL assigns uniform weight to all samples, which can be harmful if low-trust samples dominate. Therefore, a trust-weighed CORAL objective is proposed.

Let ${\overset{¯}{f}}_{i}^{s}$ be normalized trust-weighted features obtained from the trust-modulated encoder and ${\overset{¯}{f}}_{j}^{t}$ be features from target unlabeled training data. They are stacked into matrices, mathematically given as:

(22) $$F_{s}\in R^{N_{s}\times d},F_{t}\in R^{N_{t}\times d}$$

Standard CORAL uses centered covariances as given in Equations (23) and (24) and minimizes $L_{coral}$ as given in Equation (25).

(23) $$C_{s}=\frac{1}{N_{s}-1}(F_{s}-\mu_{s}^{⊤}{)}^{⊤}(F_{s}-\mu_{s}^{⊤})$$

(24) $$C_{t}=\frac{1}{N_{t}-1}(F_{t}-\mu_{t}^{⊤}{)}^{⊤}(F_{t}-\mu_{t}^{⊤})$$

(25) $$L_{coral}=∥C_{s}-C_{t}{∥}_{F}^{2}$$

Trust normalization is performed independently for source and target batches. In trust weighted CORAL, normalized trust weights ${\hat{t}}_{i}^{s}$ and ${\hat{t}}_{j}^{t}$ are incorporated and diagonal matrices are defined as given in Equation (26).

(26) $$W_{s}=\mathrm{diag}({\hat{t}}_{1}^{s},\ldots ,{\hat{t}}_{N_{s}}^{s}),W_{t}=\mathrm{diag}({\hat{t}}_{1}^{t},\ldots ,{\hat{t}}_{N_{t}}^{t})$$

In practice, features are mean-centered before applying the weighted second-order formulation Then a weighted second moment form is given in Equation (27) as:

(27) $$C_{s}^{tw}=(F_{s}-\mu_{s}{)}^{T}W_{s}\left(F_{s}-\mu_{s}\right),\;\;C_{t}^{tw}=(F_{t}-\mu_{t}{)}^{T}W_{t}\left(F_{t}-\mu_{t}\right)$$

The trust weighted CORAL loss is defined in Equation (28) as below:

(28) $$L_{twcoral}=∥C_{s}^{tw}-C_{t}^{tw}{∥}_{F}^{2}$$

This objective means that high-trust samples contribute strongly to covariance estimation, while low-trust samples contribute weakly. In milling, where target signals may include unstable transients, this weighting prevents unreliable samples from forcing the feature space to align in the wrong direction. As a result, the alignment becomes physically informed and robust to unreliable target samples.

**Step 6: Joint training and trust-aware inference**

The final model is trained end-to-end using a joint objective that combines supervised source-domain classification and unsupervised trust-weighted domain alignment. Let the complete set of trainable parameters be Θ={ψ,ω,θ}, where ψ, ω, and θ correspond to the feature encoder, classifier, and Physics Trust Network, respectively. The Physics Trust Network is not trained with explicit reliability labels. Instead, its parameters are updated through backpropagation from the same joint loss used to train the entire framework. The source-domain classification loss provides supervised task-level guidance, while the trust-weighted CORAL loss provides unsupervised adaptation guidance by using the trust scores as sample weights during covariance alignment. The training loss is defined mathematically as given in Equation (29).

(29) $$L(\Theta )=L_{cls}(\Theta )+\lambda L_{twcoral}(\Theta )$$

In Equation (29), no additional supervised trust loss is used because ground-truth reliability annotations are not available. The loss $L_{cls}$ is the supervised cross-entropy loss computed only from labeled source samples, whereas $L_{twcoral}$ is the unsupervised domain alignment loss computed using source and unlabelled target samples. Since the trust scores $t_{i}^{s}$ and $t_{j}^{t}$ affect both the trust-weighted feature representation and the weighted covariance matrices, gradients from $L_{cls}$ and $L_{twcoral}$ are propagated back to $T_{\theta }$. Therefore, $T_{\theta }$ learns reliability scores in a task-driven manner, by assigning higher influence to samples that improve class discrimination and source-target alignment, rather than by matching predefined trust labels.

During each epoch, source samples are forward through the encoder and classifier to compute $L_{cls}$. In parallel, target unlabeled samples are forward through the encoder to obtain target features and compute $L_{twcoral}$. Backpropagation updates parameters to simultaneously improve class separation on the source and reduce distribution mismatch between source and target.

After training, the model is evaluated on labeled target-test samples. For each test sample, we compute trust-weighted features and predicted probabilities as.

(30) $$\hat{y}=\mathrm{softmax}(C_{\omega }(\overset{¯}{f}))$$

In Equation (30), where $\overset{¯}{f}$ denotes the normalized trust-weighted feature, the predicted label is given as.

(31) $$\hat{y}=arg\;\underset{k}{max}\;{\hat{y}}_{k}$$

The accuracy is calculated using Equation (32) as given below.

(32) $$\mathrm{Acc}=\frac{1}{N_{test}}\sum_{m=1}^{N_{test}}I({\hat{y}}_{m}=y_{m})$$

This joint optimization strategy enables reliable fault diagnosis under cross-speed domain shifts by jointly utilizing physics-based trust modeling and distribution alignment.

Each vibration sample consisted of two vibration channels. From each sample, a 16-dimensional global physics feature vector is extracted and used as the input to the Physics Trust Network and the downstream encoder. Welch PSD is computed using a global segment length of 1024 samples. For patch-level spectral analysis, the segment length is set to min (256, patch length). The overlap is selected as min (nperseg/2, nperseg − 1), which avoided invalid overlap values for short signal patches. The frequency bands were defined as 0–500 Hz, 500–2000 Hz, and 2000–6000 Hz. The patch size was 256 samples. For permutation entropy, the global feature extraction used order = 5, delay = 1, and downsample = 5, whereas patch-level extraction used order = 4, delay = 1, and downsample = 1.

The Physics Trust Network is implemented as a fully connected multilayer perceptron with the structure 16-32-16-1. ReLU activation is applied after the first two linear layers, and sigmoid activation is used at the output layer to restrict the trust score to the range [0, 1]. The encoder used the structure 16-64-64 with ReLU activation, and the classifier is implemented as a linear layer from 64 latent features to four fault classes. The model is trained using the Adam optimizer with a learning rate of 0.001 and weight decay of 0.0001. The number of training epochs is set to 60. The domain-alignment coefficient λ in the trust-weighted CORAL objective is set to 1. All repeated experiments were conducted using the random seeds [1, 7, 21, 42, 100].

## 4. Results and Discussion

The performance of the proposed method is evaluated under cross-speed operating conditions to examine its ability to transfer diagnostic knowledge between different machine conditions. The model was trained using labeled source-domain data and unlabeled target-domain training data. Final diagnostic performance was evaluated on the held-out labeled target-test set, whose labels were used only for evaluation and not during training or domain alignment. Experimental results show that the proposed approach consistently maintains high classification accuracy and stable predictions despite speed variation. This indicates that incorporating reliability-aware learning improves the robustness of feature transfer and prevents unreliable samples from degrading adaptation performance. Compared with conventional domain adaptation methods, the learned features exhibit stronger separability and improved consistency across operating conditions, demonstrating effective handling of nonstationary behavior in milling vibration signals. The cross-condition validation was performed using four independently collected datasets. Datasets 1 and 2 correspond to separate acquisition sessions at 1320 rpm, while Datasets 3 and 4 correspond to separate acquisition sessions at 1440 rpm. Therefore, the evaluation considers domain shifts caused by both cross-speed variation and independent data acquisition under repeated nominal speed conditions. However, the present study is limited to two nominal spindle speeds, and additional speeds will be considered in future work to further evaluate generalization under wider operating conditions.

**Figure 7:**
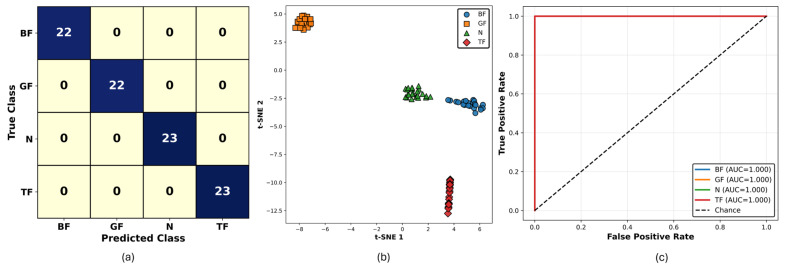
Proposed method performance under Dataset 1 at 1320 rpm as the source domain to Dataset 3 at 1440 rpm as the target domain: (**a**) confusion matrix, (**b**) t-SNE plot, and (**c**) ROC curve.

**Figure 8:**
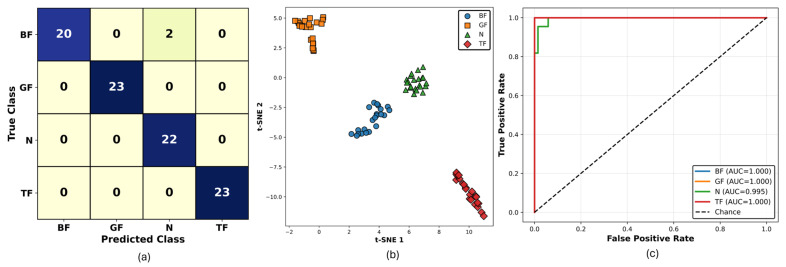
Proposed method performance under Dataset 2 at 1320 rpm as the source domain to Dataset 4 at 1440 rpm as the target domain: (**a**) confusion matrix, (**b**) t-SNE plot, and (**c**) ROC curve.

**Figure 9:**
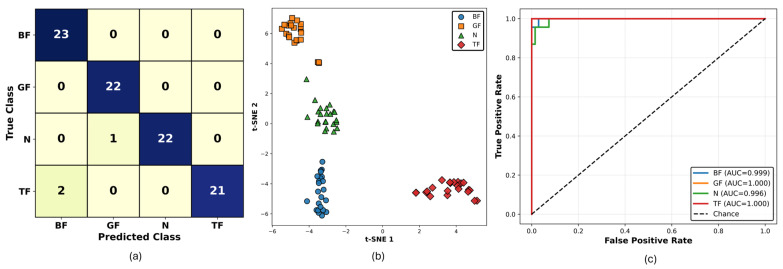
Proposed method performance under Dataset 3 at 1440 rpm as the source domain to Dataset 1 at 1320 rpm as the target domain: (**a**) confusion matrix, (**b**) t-SNE plot, and (**c**) ROC curve.

**Figure 10:**
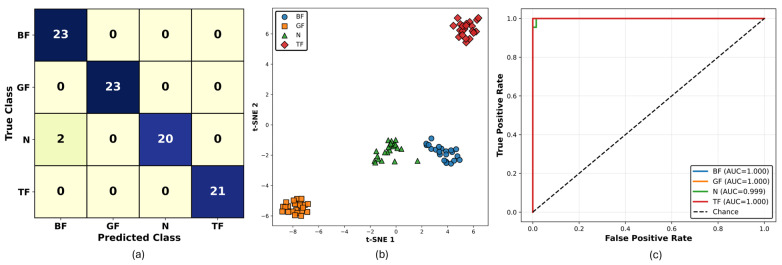
Proposed method performance under Dataset 4 at 1440 rpm as the source domain to Dataset 2 at 1320 rpm as the target domain: (**a**) confusion matrix, (**b**) t-SNE plot, and (**c**) ROC curve.

The quantitative results in Table 3 show that the proposed method maintains consistently high performance in all cases. The model achieves an average accuracy of 98.07%, with precision of 98.22%, recall of 98.04%, and F1 score of 98.04%. In particular, perfect classification is obtained for the Dataset 1 to Dataset 3 transfer, while performance remains above 96% for all other source target combinations. These results indicate that the features learned remain discriminative and transferable despite changes in operating conditions. Figure 7, Figure 8, Figure 9 and Figure 10 show the confusion matrix, t-SNE plot, and ROC curves for all four cases of the proposed method.

**Figure 11:**
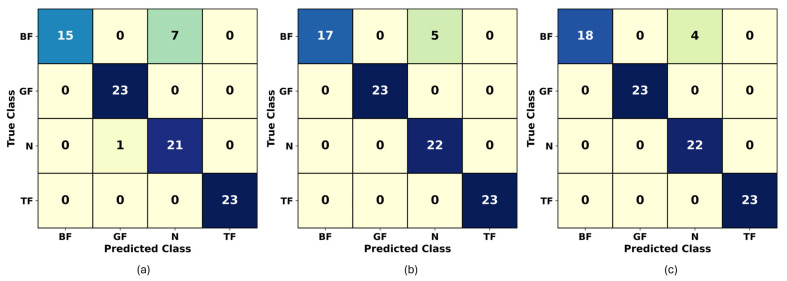
Source dataset 2 at 1320 rpm, target dataset 4 at 1440 rpm, confusion matrices for (**a**) Ablation 1, (**b**) Ablation 2, (**c**) Ablation 3.

Three ablation experiments were performed to evaluate the contribution of each component of the framework. In Ablation 1, the PTN was removed, and all samples were treated uniformly. This led to reduced prediction stability and increased overlap between fault classes in the feature space, indicating that reliability estimation plays a critical role in preventing unreliable samples from influencing adaptation. Ablation 1 can be interpreted as a standard CORAL-style baseline in which the Physics Trust Network is removed, and all samples are assigned uniform reliability weights. The performance reduction confirms that trust-guided reliability weighting improves adaptation by reducing the influence of unreliable samples. In Ablation 2, trust weighting within the feature encoder was disabled while domain alignment was retained. The resulting features exhibited lower separability and higher misclassification among similar fault categories, demonstrating the importance of selective feature emphasis during representation learning. Ablation 3 removes the trust-weighted CORAL alignment and therefore evaluates the effect of removing domain adaptation. Its reduced accuracy of 95.28% confirms that domain alignment contributes to improved cross-condition generalization. Overall, these results confirm that reliability estimation, trust-weighted representation learning, and trust-guided domain alignment jointly contribute to the improved diagnostic accuracy and robustness of the proposed method. Removing the Physics Trust Network decreases accuracy to 91.13%, showing that reliability estimation is necessary to prevent feature distortion caused by noisy samples. Disabling the trust-weighted encoder yields 93.61% accuracy, indicating that reliability must be incorporated into representation learning. Eliminating trust-guided domain alignment results in 95.28% accuracy, demonstrating that adaptation also benefits from reliability awareness during covariance alignment. The performance reductions observed in all cases confirm that each module contributes to the final performance. Confusion matrices of one of the cases of the three ablation studies are shown in Figure 11.

**Figure 12:**
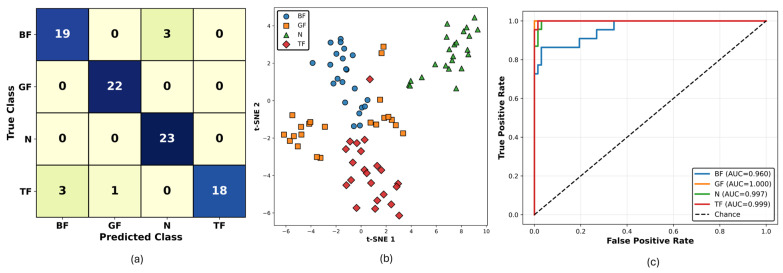
Tang et al. [36] comparison method, source dataset 3 at 1440 rpm, target dataset 1 at 1320 rpm: (**a**) confusion matrix, (**b**) t-SNE plot, (**c**) ROC curve.

The proposed method is compared with two recent states of the art domain adaptation approaches. The first baseline is proposed by Tang et al., which converts vibration signals into time frequency representations and uses an enhanced attention deep network with joint distribution alignment objectives [36]. Although this method performs effective feature alignment, it assumes that all samples contribute equally during adaptation. Under cross-speed machining conditions, unreliable vibration signals degrade the stability of feature transfer, which leads to reduced class separability. In contrast, the proposed framework evaluates the physical reliability of each sample and prioritizes consistent signals during learning, resulting in more stable adaptation and improved diagnostic accuracy. Figure 12 shows the confusion matrix, t-SNE plot and ROC curves of this comparison method.

**Figure 13:**
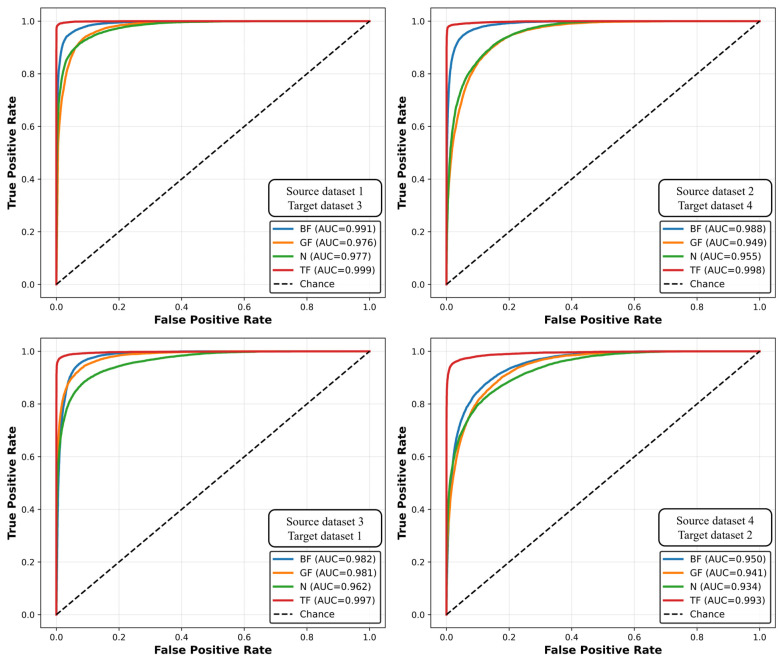
ROC curves for the Wang et al. [37] comparison method.

The second baseline is the Iterative Cluster Domain Adaptation method of Wang et al., which relies on pseudo labeling and iterative refinement to align feature distributions. While the method reduces distribution mismatch through repeated clustering and alignment, its performance strongly depends on the quality of pseudo labels [37]. In the presence of nonstationary machining dynamics, incorrect pseudo labels propagate errors and affect convergence. The proposed approach avoids this issue by guiding adaptation using reliability estimation instead of label assumptions, enabling robust knowledge transfer without iterative relabeling. Consequently, the features learned remain both discriminative and transferable across operating conditions. Figure 13 shows the ROC curves obtained for this comparison method under all cases respectively.

The method of Tang et al. achieves an average accuracy of 93.77%, whereas the ICDA method of Wang et al. reaches 86.85%. The proposed framework therefore improves accuracy by approximately 4.3% and 11.2%, respectively. Similar improvements are observed across precision, recall, and F1 score, indicating consistent classification behavior. Under cross-speed machining, vibration signals often exhibit varying amplitudes and impulsive patterns, which introduce unreliable samples. Conventional alignment methods treat all samples equally, causing incorrect feature matching between domains. By estimating the reliability of each signal, the proposed framework reduces the influence of inconsistent measurements and stabilizes feature transfer.

To further improve the interpretability of the proposed Physics Trust Network, the learned trust scores were analyzed under both cross-speed transfer directions. Since the trust score represents the estimated physical reliability of each vibration sample based on energy, spectral, impulsive, and nonlinear descriptors, physically consistent samples are expected to receive relatively higher trust values, whereas ambiguous or difficult samples are expected to receive relatively lower trust values.

**Figure 14:**
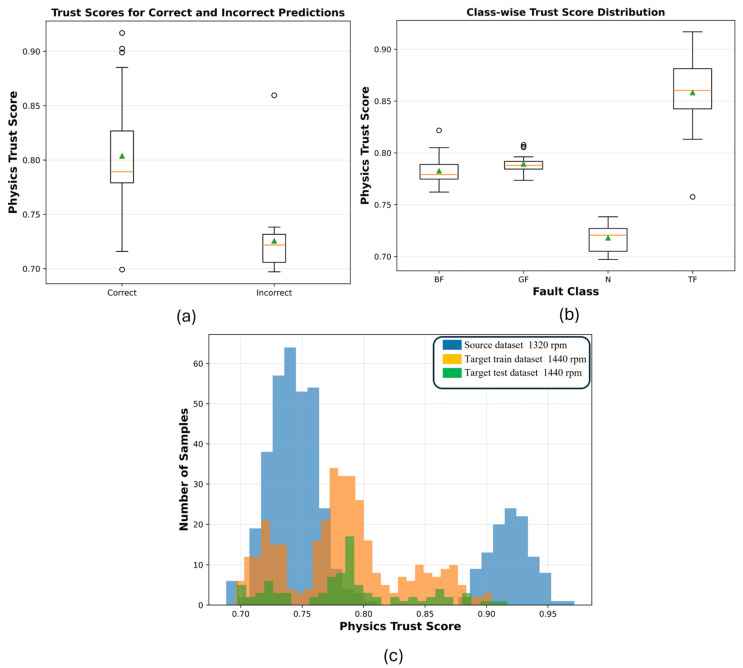
Trust-score analysis for source 1320 rpm and target 1440 rpm, (**a**) correct vs. incorrect predictions, (**b**) class-wise distributions, and (**c**) domain-wise distributions.

**Figure 15:**
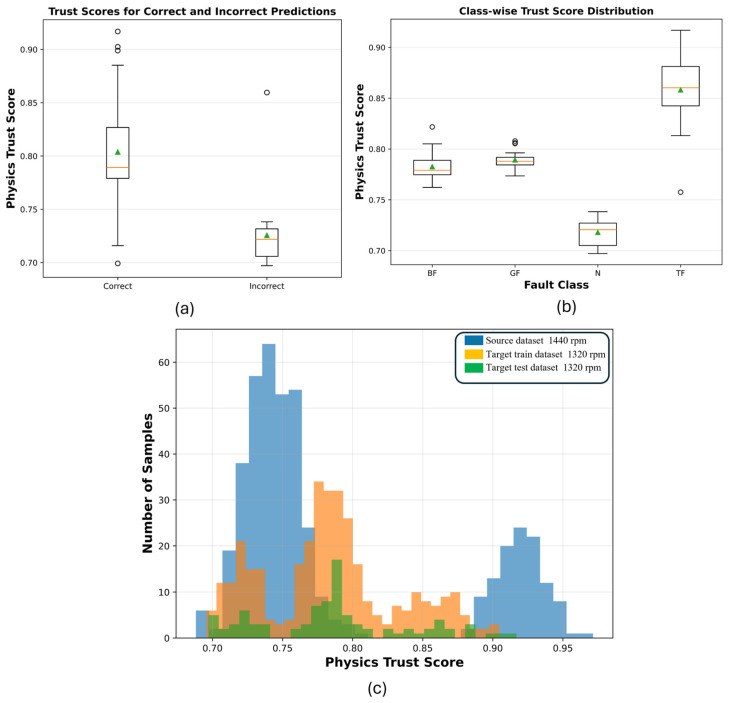
Trust-score analysis for source 1440 rpm and target 1320 rpm, (**a**) correct vs. incorrect predictions, (**b**) class-wise distributions, and (**c**) domain-wise distributions.

Figure 14 and Figure 15 show the trust-score distributions for the source domain, target-training domain, and target-test domain, together with the distributions for correctly and incorrectly classified target-test samples and the class-wise trust-score distributions. In both transfer directions, the trust scores exhibit meaningful and consistent behavior. First, correctly classified target-test samples generally have higher trust scores than incorrectly classified samples. This indicates that the proposed PTN assigns lower reliability to samples that are more ambiguous or difficult to classify, supporting its intended role as a sample-level reliability estimator.

Second, the class-wise analysis shows that different fault categories have different trust-score patterns. In the present experiments, the tool fault class exhibited comparatively higher trust values, while the normal class showed relatively lower trust values. This should not be interpreted as normal-condition samples being unreliable. Rather, it indicates that normal signals contain weaker fault-related impulsive and spectral signatures, while tool-fault signals produce more distinct and physically consistent vibration patterns. Therefore, the trust score should be interpreted as a relative reliability measure rather than a binary indicator.

Third, the domain-wise distributions show that source-domain and target-domain samples occupy partially overlapping but non-identical trust ranges in both transfer directions, indicating that the learned reliability measure remains active across different operating speeds. The smaller number of target-test samples is due to the stratified 80:20 target-domain split used in the unsupervised domain adaptation protocol, where only 20% of the target-domain samples were reserved for final evaluation.

To evaluate the practical feasibility of the proposed method, the computational cost is analyzed across all four cross-speed transfer cases. The proposed architecture is lightweight because it operates on compact 16-dimensional physics-guided feature vectors rather than directly processing high-dimensional raw vibration signals. The Physics Trust Network consists of 1089 trainable parameters, while the trust-weighted encoder and classifier contain 5508 trainable parameters. Therefore, the full model contains only 6597 trainable parameters. Across the four transfer cases, the average training time is 0.3628 ± 0.0564 s, while the average inference time is 0.001397 ± 0.000058 ms per target-test sample. These results indicate that the proposed trust-aware domain adaptation framework requires low computational resources and can perform rapid inference. Therefore, the method is computationally suitable for practical milling-machine condition monitoring applications.

To evaluate the robustness of the proposed model against noise, additional experiments were performed under different SNR levels, including 30 dB, 20 dB, and 10 dB. The model achieved average accuracies of 97.22%, 96.11%, and 94.17%, respectively, as shown in Table 4. Compared with the original average accuracy of 98.07%, the accuracy decreased by only 3.90% at 10 dB SNR. This gradual performance degradation shows that the proposed model is stable and robust under noisy conditions. Therefore, the model can maintain reliable classification performance even when the signal quality is reduced.

Overall, the results suggest that combining physics-based reliability assessment with domain adaptation improves knowledge transfer across operating conditions. The framework reduces negative transfer by limiting the impact of inconsistent signals and enables stable fault recognition under speed variation.

## 5. Conclusions

This study presents a trust-aware domain adaptation network for cross condition fault diagnosis of milling machines. The framework integrates physics-informed reliability estimation based on interpretable vibration descriptors with representation learning and domain alignment. A Physics Trust Network evaluates the reliability of each signal using physically interpretable vibration features, and the obtained trust information guides both feature learning and domain adaptation. As a result, reliable samples contribute more strongly to model training while inconsistent measurements have reduced influence, allowing the network to learn discriminative and transferable features. Experimental evaluation across multiple transfer tasks showed stable performance with an average accuracy of 98.07%, outperforming recent domain adaptation approaches. Consistent improvements were also observed in precision, recall, and F1 score. Ablation analysis further demonstrated that removing reliability estimation, reliability guided feature learning, or reliability guided alignment each reduced performance, confirming that the improvement arises from the combined effect of all modules. The results indicate that incorporating physical reliability into domain adaptation reduces negative transfer and improves robustness under varying operating conditions. The proposed strategy therefore provides a practical approach for condition monitoring when operating parameters change and labeled data are limited. Future work will investigate real time monitoring and online adaptation under continuously evolving conditions. Extending the framework to temporal trust modeling, multi sensor fusion, and remaining useful life prediction may enable a more comprehensive predictive maintenance system for industrial applications. Although the proposed framework was evaluated under cross-speed operating conditions, Cutter–Workpiece Engagement (CWE) is not explicitly varied in the present experimental setup. Future work will therefore consider datasets collected under different radial and axial depths of cut, engagement angles, and feed conditions to evaluate the robustness of the proposed method against CWE-induced distribution shifts.

## Figures and Tables

**Table 1:** Specifications of data acquisition devices.

Device	Specifications	
**Accelerometer (622b01)**	Frequency	0.40–10 kHz
	Sensitivity	100 mV/g (10.2 mV/(m/s^2^)) ± 5%
**DAQ (NI9234)**	Frequency	0–13.1 MHz
	Generator	Four analog input channels and 24-bit ADC resolution

**Table 2:** Details of dataset.

Data Set	Operating Speed	Number of Samples			
		Normal	Gear Fault	Tool Fault	Bearing Fault
**Dataset 1**	1320 rpm	116	111	112	112
**Dataset 2**	1320 rpm	111	111	106	115
**Dataset 3**	1440 rpm	112	110	112	112
**Dataset 4**	1440 rpm	111	112	112	111

**Table 3:** Performance metrics score of the proposed and comparison methods.

Method	Source Dataset	Target Dataset	Accuracy	Precision	Recall	F1 Score
**Proposed**	Dataset 1	Dataset 3	100.00%	100.00%	100.00%	100.00%
	Dataset 2	Dataset 4	97.78%	97.92%	97.73%	97.72%
	Dataset 3	Dataset 1	96.70%	96.94%	96.71%	96.68%
	Dataset 4	Dataset 2	97.78%	98.00%	97.73%	97.77%
**Average Scores**			**98.07%**	**98.22%**	**98.04%**	**98.04%**
**Ablation 1**	Dataset 1	Dataset 3	93.33%	94.83%	93.18%	93.17%
	Dataset 2	Dataset 4	91.18%	92.71%	90.91%	90.74%
	Dataset 3	Dataset 1	91.11%	91.45%	91.11%	91.19%
	Dataset 4	Dataset 2	88.89%	90.39%	88.64%	88.53%
**Average Scores**			**91.13%**	**92.35%**	**90.96%**	**90.91%**
**Ablation 2**	Dataset 1	Dataset 3	93.33%	93.54%	93.28%	93.35%
	Dataset 2	Dataset 4	94.44%	95.37%	94.32%	94.25%
	Dataset 3	Dataset 1	94.44%	94.32%	94.44%	94.28%
	Dataset 4	Dataset 2	92.22%	93.97%	91.15%	92.26%
**Average Scores**			**93.61%**	**94.30%**	**93.30%**	**93.54%**
**Ablation 3**	Dataset 1	Dataset 3	95.56%	95.60%	95.60%	95.56%
	Dataset 2	Dataset 4	95.56%	96.15%	95.45%	95.42%
	Dataset 3	Dataset 1	95.56%	95.55%	95.55%	95.55%
	Dataset 4	Dataset 2	94.44%	95.37%	94.32%	94.25%
**Average Scores**			**95.28%**	**95.67%**	**95.23%**	**95.20%**
**Tang et al. [36]**	Dataset 1	Dataset 3	97.73%	97.92%	97.73%	97.75%
	Dataset 2	Dataset 4	88.64%	92.19%	88.64%	89.11%
	Dataset 3	Dataset 1	92.13%	92.62%	92.05%	92.01%
	Dataset 4	Dataset 2	96.59%	96.74%	96.64%	96.56%
**Average Scores**			**93.77%**	**94.87%**	**93.77%**	**93.86%**
**Wang et al. [37]**	Dataset 1	Dataset 3	91.04%	91.19%	91.03%	91.07%
	Dataset 2	Dataset 4	86.09%	86.47%	86.08%	85.96%
	Dataset 3	Dataset 1	89.09%	89.49%	89.17%	89.13%
	Dataset 4	Dataset 2	81.19%	82.19%	81.38%	81.52%
**Average Scores**			**86.85%**	**87.35%**	**86.92%**	**86.92%**

**Table 4:** Noise robustness performance of the proposed model under different SNR levels.

SNR	Source Dataset	Target Dataset	Accuracy
**30 dB**	Dataset 1	Dataset 3	98.89%
	Dataset 2	Dataset 4	96.67%
	Dataset 3	Dataset 1	96.70%
	Dataset 4	Dataset 2	96.63%
**Average Scores**			**97.22%**
**20 dB**	Dataset 1	Dataset 3	97.78%
	Dataset 2	Dataset 4	95.56%
	Dataset 3	Dataset 1	95.60%
	Dataset 4	Dataset 2	95.51%
**Average Scores**			**96.11%**
**10 dB**	Dataset 1	Dataset 3	95.56%
	Dataset 2	Dataset 4	94.44%
	Dataset 3	Dataset 1	93.41%
	Dataset 4	Dataset 2	93.26%
**Average Scores**			**94.17%**

## Data Availability

The raw data supporting the conclusions of this article will be made available by the authors upon request.
